# Methanolic Extract of Dill Leaves Inhibits AGEs Formation and Shows Potential Hepatoprotective Effects in CCl_4_ Induced Liver Toxicity in Rat

**DOI:** 10.1155/2017/6081374

**Published:** 2017-01-15

**Authors:** Ebrahim Abbasi Oshaghi, Iraj Khodadadi, Fatemeh Mirzaei, Mozafar Khazaei, Heidar Tavilani, Mohammad Taghi Goodarzi

**Affiliations:** ^1^Department of Clinical Biochemistry, School of Medicine, Hamadan University of Medical Sciences, Hamadan, Iran; ^2^Student Research Committee, Kermanshah University of Medical Sciences, Kermanshah, Iran; ^3^Fertility and Infertility Research Center, Kermanshah University of Medical Sciences, Kermanshah, Iran; ^4^Research Center for Molecular Medicine, Hamadan University of Medical Sciences, Hamadan, Iran

## Abstract

The research was aimed at evaluating the antiglycation, antioxidant, and hepatoprotective properties of methanolic extract of* Anethum graveolens* (dill). The antioxidant properties, photochemical characteristics, and antiglycation effects of dill extract were measured. Carbon tetrachloride-induced hepatotoxic rats were used to show the hepatoprotective activity of dill leaves. Different concentration of dill extract (0.032, 0.065, 0.125, 0.25, 0.5, and 1 mg/mL) showed potential antioxidant ability. The extract of dill leaves significantly reduced AGEs formation and also fructosamine and protein carbonyl levels in rats' liver. Thiol groups' oxidation, amyloid cross-*β*, and protein fragmentation (*P* < 0.001) significantly reduced in treated rats. Liver damage markers significantly reduced in dill-treated animals (*P* < 0.05). Dill with potential antioxidant, antiglycation, and hepatoprotective effects can be suggested for treatment of diabetes complications.

## 1. Introduction

Free radicals are involved in many chronic and acute disorders such as diabetes, cancer, cardiovascular disease, immunosuppression, and neurological problems [1]. The detrimental effects of the free radicals can be blocked by natural antioxidants [2]. Numerous kinds of herbal medicine have studied for their antioxidant and antiradical properties [3].* Anethum graveolens* L. (dill) belongs to Apiaceae family and grows mostly in Europe, Mediterranean region, and Asia [4]. Dill is used for various purposes in many countries and traditionally used for medicinal purpose such as digestive disorders, reduction of the bad breath, and stimulation of lactation and also known as a lipid lowering, anticancer, antimicrobial, antidiabetic, antigastric irritation, anti-inflammatory, and antioxidant agent [4, 5]. Administration of dill in human and animal models had antioxidant activity and normalized blood glucose and lipid profile [4–9]. Dill also showed potential antidiabetic activity [10]. The exact antidiabetic mechanism of dill has not been recognized until now. The previous reports have not investigated all of the antioxidant indices of* Anethum graveolens*, neither its antiglycation effects. Furthermore, variance in cultivating area and the method of extraction cause different antioxidant ability [3]. Consequently, this study was planned to assess the antiglycation and oxidant scavenging as well as hepatoprotective effects of dill cultivated in Hamadan (west of Iran).

## 2. Materials and Methods

### 2.1. Extraction of Plant Materials and Phytochemical Screening

*Anethum graveolens* was prepared from Hamadan (west of Iran) and identified by our colleague in the Buali-Sina University, Hamadan, Iran. For preparation of methanolic extract, dill leaves powder was dried and crushed. Dried dill powder (100 g) was mixed with 300 mL of methanol at room temperature for 48 hours. The prepared solution was filtered and subsequently concentrated and evaporated to dryness in vacuum. The extract was kept in dark vials at −20°C until analysis [11].

### 2.2. Phytochemical Screening

Phytochemical screening was performed according to Salmanian et al. [12] and Abbasi Oshaghi et al. [13] method. Total phenolic content of methanolic extract was determined using Folin-Ciocalteu reaction. Briefly, one milligram of methanolic extract was dissolved in the reaction solution (3.8 mL of deionized water + 2 mL of 2% Na_2_CO_3_ + 100 *μ*L of 50% Folin-Ciocalteu). The prepared mixture was incubated at room temperature for 30 min and the absorbance of the sample was determined at 750 nm. Flavonoids content of dill was determined by using AlCl_3_ assay. Briefly 500 *μ*L of the dill extract (1 mg/mL in methanol) was mixed with the reaction solution (1.5 mL of 95% alcohol + 100 *μ*L of 10% AlCl_3_ + 100 *μ*L of 1 M potassium acetate + 2.8 mL of deionized water). After 40 min of incubation at room temperature the absorbance of the samples was measured at 415 nm. Total flavonols content of dill was determined by adding of 1 mg/mL dill extract to the reaction solution (200 *μ*L of 20 mg/mL AlCl_3_) + 6 mL sodium acetate solution (50 mg/mL). After 2.5 hours of incubation at room temperature the absorbance of the prepared solution was measured at 440 nm. The results were calculated per mg equivalents of gallic acid (for phenolic) and quercetin (for flavonoids and flavonols) per gram of each extract.

### 2.3. Antioxidant Activity

To measure the antioxidant activity of prepared dill extract different tests were carried out including ferric reducing antioxidant power (FRAP), DPPH radical scavenging, superoxide anion and hydrogen peroxide scavenging, metal chelating, reducing power, and nitric oxide scavenging activity, according to the previously published methods [12].

### 2.4. Glycation of BSA and Fructosamine

Glycated BSA was prepared using treatment of BSA with different concentration of fructose (200 and 500 mM) at different time periods (1, 2, 3, and 4 weeks) [14]. Aminoguanidine (AG) a known antiglycation agent was used as a positive control. After dialysis in PBS, glycated BSA formation was determined using a fluorometry method at an excitation wavelength of 440 nm and emission wavelength of 460 nm (spectrofluorometer, Jasco FP-6200) [14]. Nitroblue tetrazolium (NBT) reaction was used to measure the fructosamine level [15].

### 2.5. Thiol Group and Protein Carbonyl Content

The free thiol and carbonyl contents in glycated BSA were determined according to Adisakwattana et al.'s report [14].

### 2.6. Protein Aggregation and Fragmentation

Amyloid cross-*β* structure, which is recognized as an indicator of protein aggregation, was determined using Congo red dye [14]. The fragmentation of protein was estimated and shown using SDS-PAGE [14].

### 2.7. In Vivo Studies

#### 2.7.1. Hepatoprotective Activity

Male Wistar rats weighing 210–220 g were divided randomly into four groups (*n* = 6): (1) normal rats that received 30% CCl_4 _in olive oil (1 mL/kg body wt i.p) every 72 hours for a period of 10 days (hepatotoxic group); (2) CCl_4_ hepatotoxic induced rats that received 100 mg/kg dill extract for 10 days; (3) CCl_4 _induced hepatotoxic rats that received 300 mg/kg dill extract for 10 days; (4) normal rats that received distilled water (1 mL/kg body wt) orally for 10 days [16]. After that, the animals were anesthetized and blood was collected from their heart. All of biochemical assays were performed using commercial kits (Pars Azmun, Iran) [5]. All procedures were approved by ethics committee of Hamadan University of Medical Science, Hamadan, Iran.

#### 2.7.2. Histopathological Examination

The pieces of rats' liver were excised and then fixed in 10% formalin solution and processed by standard way. Liver sections with thickness of 5 *μ*m were stained with haematoxylin and eosin (H&E). The stained slides were evaluated under a light microscope.

### 2.8. Statistical Analysis

Data are expressed as means ± SEM of three duplicate measurements and then analyzed by SPSS package (version 16, SPSS, Inc). One way analysis of variance (ANOVA) followed by Tukey test was used to analyze the results. The *P* values less than 0.05 were regarded as statically significant.

## 3. Results

### 3.1. In Vitro Antioxidant Study

**Figure 1:**
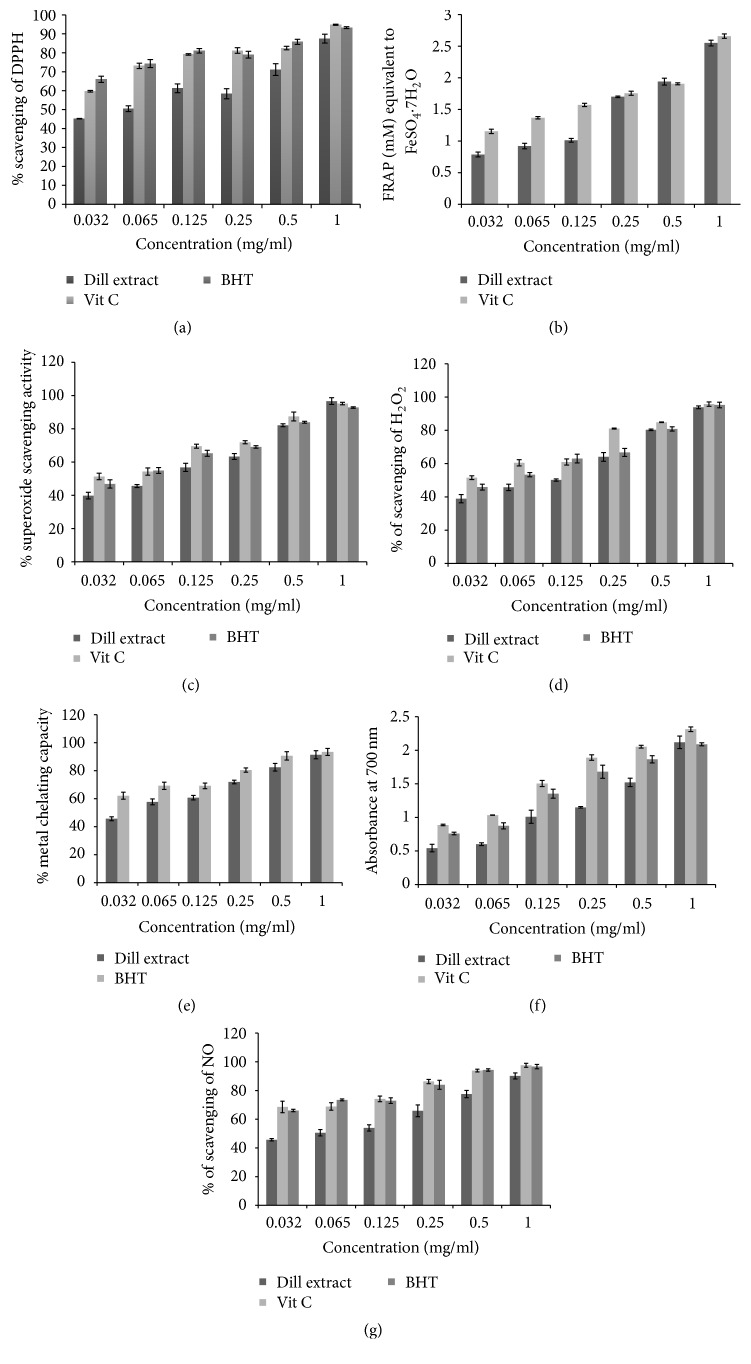
Antioxidant and antiradical activity of dill extract. Values are the average of triplicate experiments and presented as mean ± SEM. (a) DPPH radical scavenging activity. (b) FRAP assays. (c) Superoxide radical scavenging activity. (d) Hydrogen peroxide radical scavenging activity. (e) Metal chelating activity. (f) Reducing power activity. (g) Nitric oxide scavenging activity.

Dill extract showed strong DPPH radical scavenging activity in a dose dependent manner with IC50 of 0.064 mg/mL. Dill extract also had potential FRAP value and reducing power ability (Figure 1). Dill showed potential super oxide anion-, hydrogen peroxide- and NO-scavenging activity and metal chelating with IC_50_ of 0.110, 0.125 mg/mL, 0.064, and 0.056 mg/mL, respectively (Figure 1). The total phenols, flavonoids, flavonols, alkaloid, anthocyanin, tannins, and saponin contents were 176 ± 5.2, 130 ± 4.4, 121 ± 3.8, 88 ± 5.1, 46 ± 2.9, 66 ± 3.7, and 45 ± 3.2 mg/g of extract, respectively.

**Figure 2:**
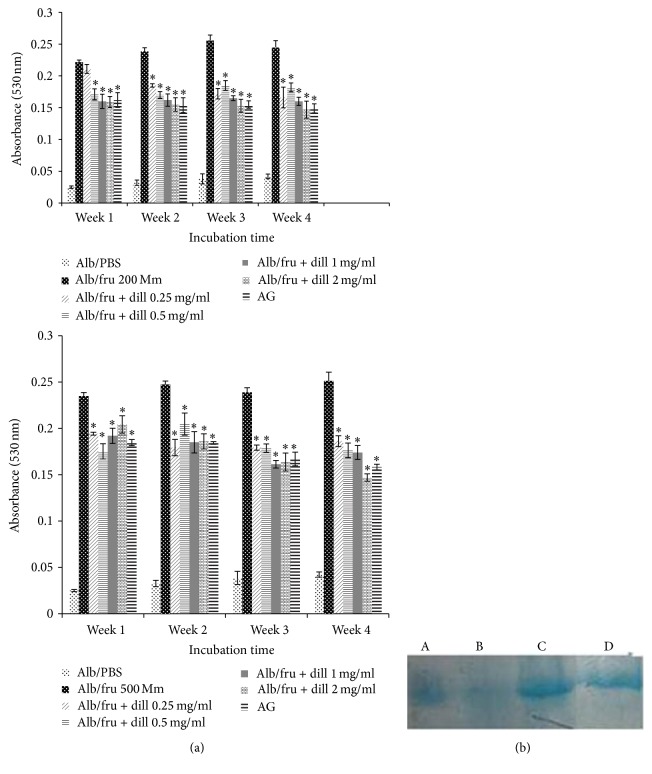
(a) The effect of dill extract on the level of protein aggregation. Data are represented as mean ± SEM (*n* = 3); ^*∗*^*P* < 0.001 compared with BSA/fructose at the same incubation time. AG: aminoguanidine. Data are represented as mean ± SEM (*n* = 3); ^*∗*^*P* < 0.001 compared with BSA/fructose at the same incubation time. (b) Protein fragmentation in BSA incubated with 200 mM fructose in the presence of Cu^+2^ ion, aminoguanidine, and dill extract for 7 days, detected by SDS-PAGE. Protein fragmentation inhibited by aminoguanidine (lane C) and dill extract (lane D) compared with BSA/fructose. A lane: 10 mg/mL BSA, B lane: 10 mg/mL BSA + 200 mM fructose, C lane: 10 mg/mL BSA + 200 mM fructose + aminoguanidine, and D lane: 10 mg/mL BSA + 200 mM fructose + dill extract.

In vitro antiglycation study of dill extract at different concentration significantly declined AGEs formation at 1, 2, 3, and 4 weeks of incubation. Dill also significantly declined fructosamine levels (Table 1) and carbonyl content (Table 2) and also inhibited thiol groups oxidation (Table 2), amyloid cross-*β* structure, and protein fragmentation rate (Figure 2).

In the in vivo study the serum levels of LDH, ALP, AST, ALT, *γ*-GT, total bilirubin, direct bilirubin, triglycerides, total cholesterol, and liver weight were significantly increased, whereas total protein, albumin, and body weight significantly reduced in CCl_4_ group. These values normalized in the animals which were pretreated with dill extract (*P* < 0.05 for all factors) (Table 3).

### 3.2. Histopathological Change in Liver

**Figure 3:**
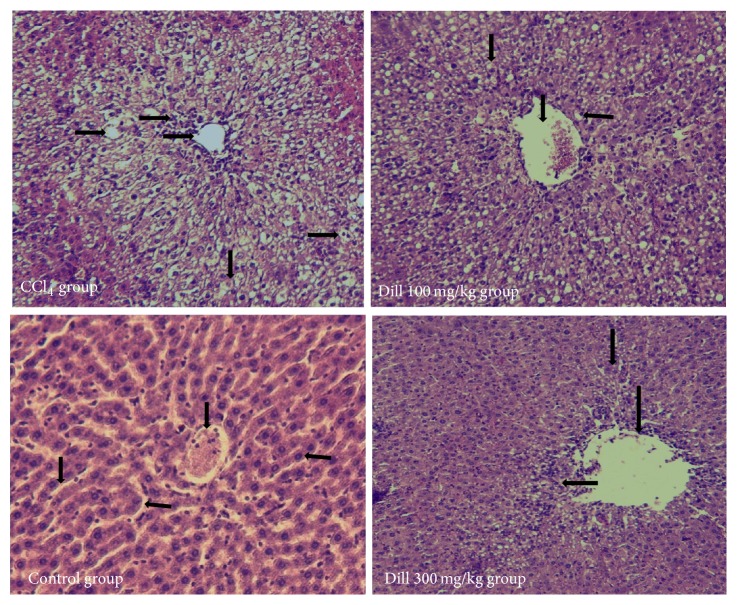
Histopathological changes in the liver of different treated animals. Histology of liver in normal group showed regular structure, while CCl_4_-treated animals show the entire damage of hepatocytes. In dill-treated animals liver damage was restored.

The microscopic analysis revealed varying degree of cellular damage from normal to severe in different treated groups (Figure 3). Liver histology in control group showed regular structure including well-organized cells sinusoidal lining and clear central vein. The CCl_4_-treated group illustrated the entire damage of hepatocytes, destruction of normal euchromatic nucleus, degeneration of central vein, fat accumulation, and foam cell formation. Also, in CCl_4_-treated group, centrilobular necrosis in most cases, various size vacuoles, and mild fibrosis were observed. These histopathological changes were repaired near to normal structure in the CCl_4_-treated animals that received 100 mg/kg and 300 mg/kg dill extract. The restoration of these changes in 300 mg/kg was more than that of CCl_4_-treated animals that received 100 mg/kg dill extract.

## 4. Discussion

In this study extract of dill leaves showed high amount of phenolic and flavonoid. Lisiewska et al. [17] found that the higher leaves of plant have higher amount of phenolics; the stem of dill has the lowest amount of phenolics. These components are able to inhibit lipid peroxidation and have useful effect in mutagenesis, carcinogenesis, atherogenesis, and thrombosis [12]. The hypolipidemic, antidiabetic, and hepatoprotective properties of the dill may be attributed to the high levels of flavonoids, which have been established to have antioxidant activity. The antioxidant properties of these agents are mostly because of their redox activities, which allow them to have different activity such as hydrogen donors, reducing metabolites, reactive oxygen species quenchers, and metal chelating. In this study relatively high amount of alkaloids was found in dill extract. Agrawal et al. [18] reported that alkaloids have potential antioxidant and hypoglycemic effect in diabetic animals. Anthocyanins also have many biological properties, including anticarcinogenic, antioxidant, and anti-inflammatory activities. Setorki et al. [19] reported that dill had moderate level of anthocyanins. Tannins also are found in dill and many researches showed their useful properties in management of diabetes complications by inhibition of oxidative stress and AGEs formation. Santos et al. [20] showed that administration of tannins markedly declined glucose and lipid levels in diabetic animals. Nakagawa and Yokozawa [21] reported that tannin inhibited AGEs formation. Saponins also were found in the dill extract and have numerous pharmacological properties such as motivation of insulin and C-peptide secretion, antioxidant activity, inhibition of AGEs formation, and also declining of diabetic nephropathy [22]. It has been reported that saponins rise permeability of the intestinal mucosal cells and increase the various nutrient absorption. Consequently, these components increased the phenolics absorption. Furthermore, these components possess antioxidant activity that involves effectiveness of the phenolics to protect against CCl_4_ induced hepatotoxicity [23]. Shyu et al. [3] reported high amounts of flavonoids, phenols, and proanthocyanidins in the ethanolic extract of* Anethum graveolens* flower.

Our results showed potential antioxidant activity for dill in different tests. The stable free radical of DPPH and FRAP value generally are used to evaluate plant antioxidant ability by working as hydrogen donors or free radical scavengers [12]. Bahramikia et al. [1] reported that water extract fraction of dill had significant DPPH scavenging activity. Superoxide anion involves the development of other ROS including hydroxyl radical, singlet oxygen, and hydrogen peroxide (H_2_O_2_), which stimulates oxidation of proteins, lipids, and DNA. Studies showed that antioxidant effects of some flavonoids are efficient, predominantly by O_2_^−^ scavenging activity [12, 24]. Among the different species of metal ions, iron (II) is known as the strong prooxidant [12]. Iron chelating activity of dill extract is similar to ascorbic acid and BHT [24]. The reducing power of the agents could serve as a remarkable indicator of their antioxidant activity; therefore, the effectiveness of certain antioxidant agents is famous to be related to high reducing power activity. The reducing power of dill maybe related to its ability to donate hydrogen [24]. NO is a reactive compound which reacts with oxygen and leads to formation of oxidized form of nitrogen [24]. Dill showed potential NO scavenging activity in a dose dependent manner.

Fructose and its metabolites are supposed to be important precursors of AGEs formation in the intracellular condition [25]. Consumption of aminoguanidine (AG) has sufficient effects on diabetic complications; however, it has some harmful side effects such as hepatotoxicity and drug resistance [14]. Therefore, administration of natural products with antiradical and antioxidant effects and low side effects makes them good candidates in treatment of diabetes complications. The dill ability to reduce AGEs formation might contribute to its antioxidant activity [26]. In agreement with Bahramikia et al. [1] studies, we showed that dill has potential antioxidant activity. The metal chelating activity has been shown to be one of the major mechanisms for antiglycation property [25]. In this study, dill extract at different concentration significantly showed iron chelating activity. Presence of tannins in dill extract plays a critical role in treatment of diabetes complications through inhibition of oxidative stress and AGEs formation [20]. Nakagawa and Yokozawa [21] showed that green tea contains high amounts of tannins which significantly inhibits AGE formation. The other mechanism suggested for antiglycation activity is a break of the cross-linking constitution in the AGEs, reducing the carbonyl groups, Amadori products, or Schiff's bases and also reduction of the late-stage Amadori products [26]. Declining of fructosamine levels has beneficial approach to delay vascular complications of diabetes [25]. Our findings indicate dill extract significantly reduced fructosamine levels.

Some studies reported that administration of aqueous extract of dill declined fasting blood glucose in animal models. Mobasseri et al. [8] showed dill normalized lipid profiles and insulin sensitivity in diabetic patients. We previously showed that administration of dill in diabetic animals led to normalized blood glucose, lipid profile, and antioxidant capacity [26, 27]. Rashid Lamir et al. [9] also established that aerobic training with usage of dill significantly increased HDL-C levels and declined blood glucose and LDL/HDL ratio in diabetic women.

Increasing of carbonyl content and declining of free thiol groups are directly reflected to oxidation of protein [26]. Our study showed that dill extract markedly reduced protein carbonyl content and also increased thiol groups. Aggregation of protein causes amyloid cross-*β* structure formation which can be determined via reaction with Congo red dye. Dill extracts markedly inhibited protein aggregation. The aggregated protein is able to produce amyloid cross-*β* structure and subsequently change stability of protein and its structure [28]. Fragmentation of BSA in the presence of fructose and Cu^2+^was reduced significantly by dill extract. Incubation of glycated BSA with Cu^2+^ is accompanied by the decline of protein-bound glucose, showing that fragmentation of protein occurred at the expense of BSA-bound glucose [29]. Sakai et al. [29] showed that incubation of protein with fructose and Cu^2+^markedly increased BSA fragmentation, while AG inhibited this process.

The administration of methanol extract of dill protects the liver from induced damage by CCl_4 _as manifested by improvement of biochemical factors. The hepatoprotective mechanism of dill is unclear but may be related to presence of many phytoconstituents and lipid peroxidation inhibitors. The hepatotoxicity induced by CCl_4 _is correlated to production of ^•^CCl_3_, an active metabolite; this is displayed by marked increase in the serum liver enzymes such as AST, ALT, and ALP [23]. In this study the biochemical factor that was measured for liver function was AST, ALT, LDH, GGT, bilirubin, albumin, and total protein. Serum transferases (ALT and AST) are accepted as sensitive markers, strongly related to liver toxicity and damage [27]. Thuppia et al. [30] showed that ethanolic extract of dill has a hepatoprotective activity by declining the AST and ALT levels on acetaminophen-induced hepatic damage in rats. In this study treating the rats with dill extracts especially at the dose of 300 mg/kg did cause significant reduction on both ALT and AST levels. Actually, this extract normalized liver function test in CCl_4_ induced liver toxicity. The existence of high amount of phenolic and flavonoid in dill extract elucidates its free radical scavenging properties and probably its in vivo effect on liver function [23].

Liver is known as the main source of serum protein synthesis especially albumin [23]. We showed significant reduction in total protein and albumin by CCl_4_, which is consequently revealed the decline in protein synthesis in the liver through necrosis. While, in our experiments, treatment of rat with dill at the dose of 100 and 300 mg/kg normalized these factors. Our results are also reinforced by Rabeh and Aboraya [31] who reported that* Anethum graveolens* or fennel oil and their mixtures have a significant hepatoprotective effect against CCl_4_ induced liver toxicity. They showed that treatment of hepatotoxic rats by dill oil markedly reduced ALT, AST, ALP, and blood lipids and also increased total protein and albumin. Oral uptake of dill at doses of 100 and 300 mg/kg significantly decreased the triglycerides and total cholesterol levels. Reduction at dose of 300 mg/kg was more significant. Our result was in agreement with Yazdanparast and Bahramikia [32], Koppula and Choi [6], Thuppia et al. [30], Hajhashemi and Abbasi [33], Madani et al. [5], and other studies [34] as formerly stated that dill significantly reduced blood lipids and liver enzymes.

Histological analysis (Figure 3) also was correlated with biochemical factors. Histological analysis of hepatotoxic liver with CCl_4_ displays major morphological changes. Nevertheless, in rats treated with dill extract at the doses of 100 and 300 mg/kg, the severity of liver damage was reduced significantly, indicating its potential hepatoprotective properties. Our data were similar to the findings of Thuppia et al. [30], Rabeh and Aboraya [31], and Tamilarasi et al. [35] and also our previous results [36] that showed dill oil, dill ethanolic extract, crude powder of dill, and dill tablet have potential antioxidant and hepatoprotective effects on in rats.

## 5. Conclusion

Extract of dill leaves showed potential antioxidant, antiglycation, and hepatoprotective activities. According to the findings dill can be suggested as a good candidate for healing of diabetes complication and liver toxicity.

## Figures and Tables

**Table 1:** The effect of dill extract on AGE and fructosamine formation.

Experimental groups	AGE formation (arbitrary unit)				Fructosamine levels (mmol/mg protein)			
	Week 1	Week 2	Week 3	Week 4	Week 1	Week 2	Week 3	Week 4
*BSA/Fru 500 mM*	*95.22 ± 6.64*	*100.93 ± 7.35*	*128.15 ± 6.37*	*141.09 *± *13.1*	*3.46 ± 0.070*	*3.36 ± 0.06*	*3.64 ± 0.066*	*3.95 ± 0.063*
+Dill 0.25 mg/ml	38.87 ± 4.56^*∗*^	40.08 ± 7.15^*∗*^	44.91 ± 6.32^*∗*^	56.76 ± 8.03^*∗*^	3.37 ± 0.036	2.95 ± 0.055^*∗*^	3.03 ± 0.028^*∗*^	3.43 ± 0.005^*∗*^
+Dill 0.5 mg/ml	36.49 ± 6.81^*∗*^	39.78 ± 6.31^*∗*^	47.94 ± 7.28^*∗*^	54.65 ± 4.49^*∗*^	2.68 ± 0.105^*∗*^	2.86 ± 0.066^*∗*^	3.04 ± 0.063^*∗*^	3.39 ± 0.008^*∗*^
+Dill 1 mg/ml	28.51 ± 4.20^*∗*^	36.87 ± 5.35^*∗*^	35.32 ± 6.19^*∗*^	44.46 ± 7.33^*∗*^	2.62 ± 0.061^*∗*^	2.71 ± 0.089^*∗*^	2.83 ± 0.014^*∗*^	3.19 ± 0.020^*∗*^
+Dill 2 mg/ml	23.73 ± 3.01^*∗*^	31.64 ± 4.40^*∗*^	41.63 ± 2.98^*∗*^	47.07 ± 3.45^*∗*^	2.70 ± 0.105^*∗*^	2.75 ± 0.080^*∗*^	2.64 ± 0.008^*∗*^	2.97 ± 0.026^*∗*^
+AG 2 mg/ml	37.23 ± 5.51^*∗*^	45.56 ± 4.35^*∗*^	49.45 ± 4.02^*∗*^	55.82 ± 2.90^*∗*^	2.65 ± 0.081^*∗*^	2.74 ± 0.043^*∗*^	2.54 ± 0.139^*∗*^	2.81 ± 0.010^*∗*^
*BSA/Fru 200 mM*	*98.07 ± 4.74*	*109.77 ± 8.30*	*133.54 ± 9.14*	*151.46 ± 5.41*	*2.33 ± 0.05*	*2.54 ± 0.01*	*2.77 ± 0.08*	*2.95 ± 0.02*
+Dill 0.25 mg/ml	44.43 ± 5.96^*∗*^	49.63 ± 5.61^*∗*^	53.80 ± 6.85^*∗*^	70.32 ± 8.73^*∗*^	2.36 ± 0.07	2.03 ± 0.05^*∗*^	2.10 ± 0.02^*∗*^	2.50 ± 0.01^*∗*^
+Dill 0.5 mg/ml	37.05 ± 5.62^*∗*^	45.01 ± 4.28^*∗*^	50.83 ± 5.58^*∗*^	64.54 ± 5.30^*∗*^	1.71 ± 0.09^*∗*^	1.94 ± 0.06^*∗*^	2.11 ± 0.06^*∗*^	2.46 ± 0.01^*∗*^
+Dill 1 mg/ml	33.40 ± 6.06^*∗*^	47.10 ± 5.54^*∗*^	42.21 ± 4.15^*∗*^	50.02 ± 4.44^*∗*^	1.72 ± 0.06^*∗*^	1.79 ± 0.08^*∗*^	1.90 ± 0.01^*∗*^	2.26 ± 0.02^*∗*^
+Dill 2 mg/ml	32.95 ± 5.28^*∗*^	41.21 ± 5.50^*∗*^	45.70 ± 5.47^*∗*^	43.29 ± 5.22^*∗*^	1.80 ± 0.10^*∗*^	1.83 ± 0.08^*∗*^	1.71 ± 0.01^*∗*^	2.04 ± 0.02^*∗*^
+AG 2 mg/ml	51.22 ± 4.91^*∗*^	53.63 ± 5.55^*∗*^	52.05 ± 5.22^*∗*^	59.94 ± 4.10^*∗*^	1.76 ± 0.08^*∗*^	2.20 ± 0.06^*∗*^	2.09 ± 0.04^*∗*^	2.29 ± 0.01^*∗*^
BSA/PBS	20.73 ± 2.43^*∗*^	20.00 ± 2.21^*∗*^	26.97 ± 5.69^*∗*^	29.20 ± 4.52^*∗*^	0.15 ± 0.01^*∗*^	0.16 ± 0.01^*∗*^	0.17 ± 0.01^*∗*^	0.20 ± 0.01^*∗*^

^*∗*^ *P* < 0.01 when compared to BSA/fructose at the same incubation time.

**Table 2:** The effect of dill extract on the thioland carbonyl group.

Experimental groups	Thiol group (nmol/mg protein)				Carbonyl group (nmol/mg protein)			
	Week 1	Week 2	Week 3	Week 4	Week 1	Week 2	Week 3	Week 4
*BSA/Fru 500 mM*	*2.24 ± 0.01*	*1.69 ± 0.08*	*1.35 ± 0.02*	*0.84 ± 0.05*	*2.55 ± 0.06*	*2.68 ± 0.12*	*3.21 ± 0.06*	*3.39 ± 0.10*
+Dill 0.25 mg/ml	2.29 ± 0.08	2.14 ± 0.09^*∗*^	1.98 ± 0.03^*∗*^	1.56 ± 0.06^*∗*^	2.22 ± 0.11^*∗*^	2.03 ± 0.05^*∗*^	2.12 ± 0.03^*∗*^	2.53 ± 0.02^*∗*^
+Dill 0.5 mg/ml	2.54 ± 0.02^*∗*^	2.32 ± 0.02^*∗*^	2.17 ± 0.09^*∗*^	1.76 ± 0.07^*∗*^	2.22 ± 0.09^*∗*^	2.02 ± 0.06^*∗*^	2.11 ± 0.05^*∗*^	2.52 ± 0.01^*∗*^
+Dill 1 mg/ml	2.4 ± 0.07^*∗*^	2.48 ± 0.06^*∗*^	2.39 ± 0.07^*∗*^	1.41 ± 0.03^*∗*^	1.72 ± 0.05^*∗*^	1.79 ± 0.08^*∗*^	1.92 ± 0.01^*∗*^	2.29 ± 0.02^*∗*^
+Dill 2 mg/ml	2.76 ± 0.02^*∗*^	2.62 ± 0.02^*∗*^	2.34 ± 0.05^*∗*^	2.40 ± 0.09^*∗*^	1.80 ± 0.10^*∗*^	1.83 ± 0.07^*∗*^	1.73 ± 0.01^*∗*^	2.07 ± 0.02^*∗*^
+AG 2 mg/ml	2.47 ± 0.06^*∗*^	2.35 ± 0.01^*∗*^	2.12 ± 0.04^*∗*^	1.79 ± 0.09^*∗*^	1.97 ± 0.09^*∗*^	2.03 ± 0.02^*∗*^	2.14 ± 0.01^*∗*^	2.37 ± 0.09^*∗*^
*BSA/Fru 200 mM*	*1.99 ± 0.05*	*1.7 ± 0.05*	*1.52 ± 0.14*	*0.90 ± 0.05*	*2.26 ± 0.01*	*2.57 ± 0.03*	*2.58 ± 0.10*	*3.32 ± 0.02*
+Dill 0.25 mg/ml	2.37 ± 0.04^*∗*^	2.20 ± 0.05^*∗*^	2.09 ± 0.05^*∗*^	1.75 ± 0.01^*∗*^	2.27 ± 0.05	2.08 ± 0.04^*∗*^	2.13 ± 0.06^*∗*^	2.90 ± 0.03^*∗*^
+Dill 0.5 mg/ml	2.48 ± 0.07^*∗*^	2.36 ± 0.09^*∗*^	2.16 ± 0.07^*∗*^	1.67 ± 0.10^*∗*^	1.72 ± 0.03^*∗*^	1.93 ± 0.01^*∗*^	2.20 ± 0.05^*∗*^	2.99 ± 0.02^*∗*^
+Dill 1 mg/ml	2.66 ± 0.03^*∗*^	2.49 ± 0.09^*∗*^	2.48 ± 0.03^*∗*^	1.54 ± 0.02^*∗*^	1.73 ± 0.05^*∗*^	1.75 ± 0.06^*∗*^	2.06 ± 0.01^*∗*^	2.71 ± 0.07^*∗*^
+Dill 2 mg/ml	2.60 ± 0.14^*∗*^	2.50 ± 0.12^*∗*^	2.42 ± 0.06^*∗*^	2.31 ± 0.04^*∗*^	1.81 ± 0.09^*∗*^	1.79 ± 0.03^*∗*^	1.78 ± 0.04^*∗*^	2.65 ± 0.09^*∗*^
+AG 2 mg/ml	2.55 ± 0.07^*∗*^	2.35 ± 0.01^*∗*^	2.25 ± 0.13^*∗*^	1.87 ± 0.01^*∗*^	1.63 ± 0.07^*∗*^	2.06 ± 0.01^*∗*^	2.12 ± 0.10^*∗*^	2.38 ± 0.04^*∗*^
BSA/PBS	2.73 ± 0.06^*∗*^	2.63 ± 0.07^*∗*^	2.53 ± 0.04^*∗*^	2.51 ± 0.09^*∗*^	0.21 ± 0.05^*∗*^	0.25 ± 0.06^*∗*^	0.23 ± 0.01^*∗*^	0.24 ± 0.02^*∗*^

^*∗*^ *P* < 0.01 when compared to BSA/fructose at the same incubation time.

**Table 3:** The effect of dill extract on biochemical factors.

Biochemical factors	CCl_4_-treated	Dill (100 mg/kg) + CCl_4_	Dill (300 mg/kg) + CCl_4_	Normal group
LDH (U/l)	196.50 ± 2.29	132.33 ± 2.02^*∗*^	113.83 ± 4.7^*∗*^	103.00 ± 5.5^*∗*^
ALP (U/l)	230.17 ± 6.17	181.67 ± 3.50^*∗*^	145.67 ± 5.11^*∗*^	154.00 ± 0.54^*∗*^
AST (U/l)	273.83 ± 8.47	203.33 ± 4.43^*∗*^	109.00 ± 3.34^*∗*^	98.17 ± 3.79^*∗*^
ALT (U/l)	239.00 ± 5.31	104.50 ± 2.02^*∗*^	78.50 ± 7.48^*∗*^	54.33 ± 2.69^*∗*^
*γ*-GT (U/l)	5.45 ± 0.611	3.23 ± 0.24^≠^	2.88 ± 0.36^≠^	1.32 ± 0.12^*∗*^
Total bilirubin (mg/dl)	3.01 ± 0.14	1.78 ± 0.12^*∗*^	1.49 ± 0.08^*∗*^	0.85 ± 0.04^*∗*^
Direct bilirubin (mg/dl)	1.01 ± 0.08	0.89 ± 0.20^*¥*^	0.51 ± 0.04^≠^	0.30 ± 0.03^*∗*^
Total protein (mg/dl)	5.47 ± 0.30	6.00 ± 0.24	6.25 ± 0.11^≠^	6.46 ± 0.08^≠^
Albumin (mg/dl)	2.96 ± 0.14	3.36 ± 0.07^*¥*^	3.49 ± 0.09^≠^	3.52 ± 0.07^≠^
Triglyceride (mg/dl)	121.83 ± 5.26	111.50 ± 4.37	99.67 ± 6.69^*¥*^	84.16 ± 1.83^*∗*^
Total cholesterol (mg/dl)	110.83 ± 2.78	88.16 ± 7.10^*¥*^	71.16 ± 6.10^≠^	75.16 ± 7.56^≠^
Body weight (g)	195.67 ± 4.54	224.00 ± 1.93^*∗*^	220.50 ± 1.82^*∗*^	223.33 ± 1.34^*∗*^
Liver weight (g)	4.17 ± 0.11	3.37 ± 0.10^≠^	3.42 ± 0.14^≠^	3.34 ± 0.18^≠^

Data are represented as mean ± SEM (*n* = 6); ^*¥*^*P* < 0.05, ^≠^*P* < 0.01, and ^*∗*^*P* < 0.001 compared with CCl_4_-treated rats.
